# Evaluation of objective nutritional indices as predictors of renal progression in IgA nephropathy patients

**DOI:** 10.3389/fnut.2026.1773914

**Published:** 2026-05-13

**Authors:** Wen Zhou, Bixia Yang, Kezhi Zhou, Liqin Cui, Liang Wang, Min Yang, Yan Yang

**Affiliations:** 1 Department of Nephrology, The Third Affiliated Hospital of Soochow University, Changzhou, China; 2 Department of Nephrology, The Affiliated BenQ Hospital of Nanjing Medical University, Nangjing, China; 3 Changzhou Medical Center, Nanjing Medical University, Changzhou, China; 4 Department of Nephrology, Wuxi People’s Hospital, Wuxi, China

**Keywords:** controlling nutritional status score, IgA nephropathy, nutritional indicators, prognostic nutritional index, renal outcomes

## Abstract

**Background and Aims:**

Immunoglobulin A nephropathy (IgAN) is a clinical and pathological syndrome with heterogenous manifestation and progression. The prognostic nutritional index (PNI) and the controlling nutritional status (CONUT) score, indicators of nutritional status and systemic inflammation, are associated with poor prognosis in dialysis patients. This study was aimed to investigate the predictive value of the objective nutritional indices (PNI and CONUT) for renal progression in IgAN patients.

**Methods:**

A multicenter retrospective study was conducted in biopsy-proven IgAN patients. Baseline characteristics were obtained within 1 week before renal biopsy. The renal composite endpoint comprised an estimated glomerular filtration rate (eGFR) decline >50%, a doubling of baseline serum creatinine, or the occurrence of end stage kidney disease (ESKD). The receiver operating characteristic curve analysis was conducted to determine the optimal cut-off value of PNI and CONUT. The Kaplan–Meier curve estimated the cumulative renal-survival rate. Univariate and multivariate Cox regression models were preformed to investigate the association between objective nutritional indices and renal outcomes.

**Results:**

A total of 659 IgAN patients participated in this study. During a median follow-up period of 45 months, 68 patients (10.32%) achieved the composite endpoint. The Kaplan–Meier curve revealed that renal-survival rate was significantly higher in high PNI group (PNI > 46.5; *p* < 0.001) and low CONUT group (CONUT ≤2; *p* < 0.001). Even after adjustment of traditional risk factors, including sex, age, mean arterial pressure, hemoglobin, uric acid, eGFR, triglycerides, 24-h urinary protein, E score, T score, and treatment with ACEI/ARB, low PNI [hazard ratio (HR) = 2.514, 95% confidence intervals (CI) = 1.212–5.215, *p* = 0.013] and high CONUT (HR = 2.152, 95% CI = 1.087–4.259, *p* = 0.028) remained as independent risk factors for poor renal outcomes.

**Conclusion:**

This study suggested that low PNI and high CONUT were significantly and independently correlated with poor prognosis in patients with IgAN at CKD stages 1–4. The PNI and CONUT are inexpensive and straightforward indicators to help clinicians improve IgAN management.

## Introduction

Immunoglobulin A nephropathy (IgAN) is the most common primary glomerular disease worldwide and represents one of the leading causes of chronic kidney disease (CKD) and end-stage kidney disease (ESKD) (1). The clinical presentation of IgAN is highly heterogeneous, ranging from asymptomatic haematuria and/or proteinuria to nephrotic syndrome, intermittent episodes of visible haematuria triggered by respiratory tract or gastrointestinal tract infection, rapidly progressive glomerulonephritis, and progressive kidney function decline (2). The prevalence of IgAN exhibits a West-to-East increasing gradient, with the highest frequency observed in East Asia, accounting for approximately 40–50% of all native kidney biopsies (1). Long-term follow-up studies have indicated that approximately 25–50% of IgAN patients progress to ESKD within 20 years after diagnosis (3, 4). Consequently, it is imperative to identify high-risk IgAN patients early and to initiate precise, timely clinical interventions aimed at slowing disease progression, lowering the incidence of ESKD, and improving long-term outcomes.

Several factors assessed at renal biopsy, including proteinuria, severe histologic grading, hypertension and impaired renal function, have been established as significant predictors of IgAN prognosis (5–8). The international prediction tool for IgAN developed by Barbour et al. (6) has been incorporated into the 2025 Kidney Disease: Improving Global Outcomes (KDIGO) clinical practice guideline for the management of IgAN (9). This model integrates multiple prognostic factors, including age, race, estimated glomerular filtration rate (eGFR), proteinuria, medication history, blood pressure at biopsy, and the Oxford Classification (MEST-score) (10), to predict clinical outcomes. In addition, malnutrition has also been demonstrated to be a significant risk factor for poor prognosis in IgAN patients (11), an association thought to be mediated by chronic inflammation, impaired intestinal mucosal barrier function, and immune dysregulation (12, 13).

In recent years, emerging tools such as the prognostic nutritional index (PNI) and the controlling nutritional status (CONUT) score have garnered increasing attention. By integrating various nutrition-related biomarkers, including serum albumin, lymphocyte count, and total cholesterol, these tools provide a more comprehensive assessment of nutritional status and have established prognostic utility in dialysis patients (14, 15). However, the relevance of these nutritional indices to prognosis in IgAN has not been thoroughly investigated. Therefore, this study will investigate the association of PNI and CONUT score with renal prognosis in IgAN to evaluate their utility for the early identification of high-risk individuals and to inform clinical decision-making strategies.

## Materials and methods

### Study design and population

We conducted a multicentre retrospective study in Changzhou First People’s Hospital and Wuxi People’s Hospital. The study included primary biopsy-proven IgAN patients diagnosed between January, 2016 and December, 2021. The exclusion criteria included: (1) age <15 years old; (2) eGFR <15 mL/min/1.73m^2^ at the time of renal biopsy; (3) less than 8 glomeruli in a renal tissue section; (4) secondary IgA nephropathy such as systemic lupus erythematosus, chronic hepatitis, and purpura; (5) concomitant with infectious diseases, malignancies, or severe organic diseases including refractory end-stage heart failure, severe arrhythmia, aortic aneurysm/dissection, severe respiratory dysfunction, severe cerebrovascular disease etc.; (6) missing required data to calculate the CONUT score and PNI. The study adhered to STROBE guidelines and obtained ethical approval from the Ethics Committee of the Changzhou First People’s Hospital and Wuxi People’s Hospital in accordance with the principles of the Declaration of Helsinki. Written informed consent was obtained from all patients.

### Data collections

We obtained demographics such as age, gender, comorbidities, diastolic blood pressure and systolic blood pressure from Electronic Medical Record System. Laboratory data including 24 h urinary protein, urine red blood cell counts, hemoglobin, lymphocyte count, albumin, globulin, fasting blood glucose, serum creatine, blood urea nitrogen (BUN), uric acid, cholesterol, triglyceride, and serum complement level were obtained within 1 week before renal biopsy. Serum albumin was measured using the bromocresol green method. Comprehensive supportive care [angiotensin-converting enzyme inhibitors (ACEI) or angiotensin receptor blockers (ARB)] and immunosuppressive therapy were given according to clinical and pathological severity. Mean arterial pressure (MAP) was calculated as diastolic blood pressure plus 1/3 of the pulse pressure. Chronic Kidney Disease Epidemiology Collaboration (CKD-EPI) equation is used to calculate eGFR (16). PNI is determined by the formula: 10 × serum albumin (g/dL) + 0.005 × total lymphocyte count (/mL) (17). The CONUT score is derived from the following items: lymphocyte count, serum albumin and total cholesterol (Supplementary Table S1) (18). The updated Oxford Classification for IgAN was given by two experienced renal pathologists who were blinded to the clinical data. Discrepancies were resolved by discussion or consultation of a third renal pathologist. Global sclerosis referred to a glomerular lesion exhibiting scarring or hyaline deposition that affected ≥ 50% of the glomerular area. Direct immunofluorescence for IgG, IgA, IgM, C1q, and C3 was routinely performed on 5-μm frozen sections.

### Clinical outcomes and follow-up

The renal composite endpoint comprised the first occurrence of any of the following: (1) doubling of the serum creatinine level from baseline; (2) decline in eGFR of >50%; (3) progression to ESKD, including an eGFR < 15 mL/min/1.73m^2^, initiation of renal replacement therapy (dialysis), or kidney transplantation. Patient follow-up was conducted through the hospital medical record review and telephone interviews until the onset of renal composite endpoint or April 30, 2024, whichever occurred first.

### Statistical analyses

Continuous variables with normal distribution were compared using the independent Student’s t-test, whereas non-normal distribution variables were analyzed with the Mann–Whitney U test or Kruskal-Wallis H test. Categorical variables were compared using the Chi-square test or Fisher’s exact test. The receiver operating characteristic (ROC) curve analysis was accessed to evaluate the utility of baseline PNI and CONUT scores in discriminating renal outcomes in IgAN patients. The area under the ROC curve (AUC) was calculated to identify the optimal cut-off values for each score. Kaplan–Meier survival analysis was used to compare cumulative renal survival rates. Correlations between PNI or CONUT score and selected variables were analyzed using Spearman’s rank correlation coefficients. To identify factors influencing renal outcomes, univariate and multivariate Cox regression models were employed. Variables significant at *p* < 0.05 in the univariate analysis were incorporated into the final multivariate model. We performed subgroup analyses with forest plots to assess the consistency of the effects of baseline categorical PNI and CONUT scores across predefined subgroups. A two-sided *p*-value < 0.05 was considered statistically significant.

## Results

**Figure 1:**
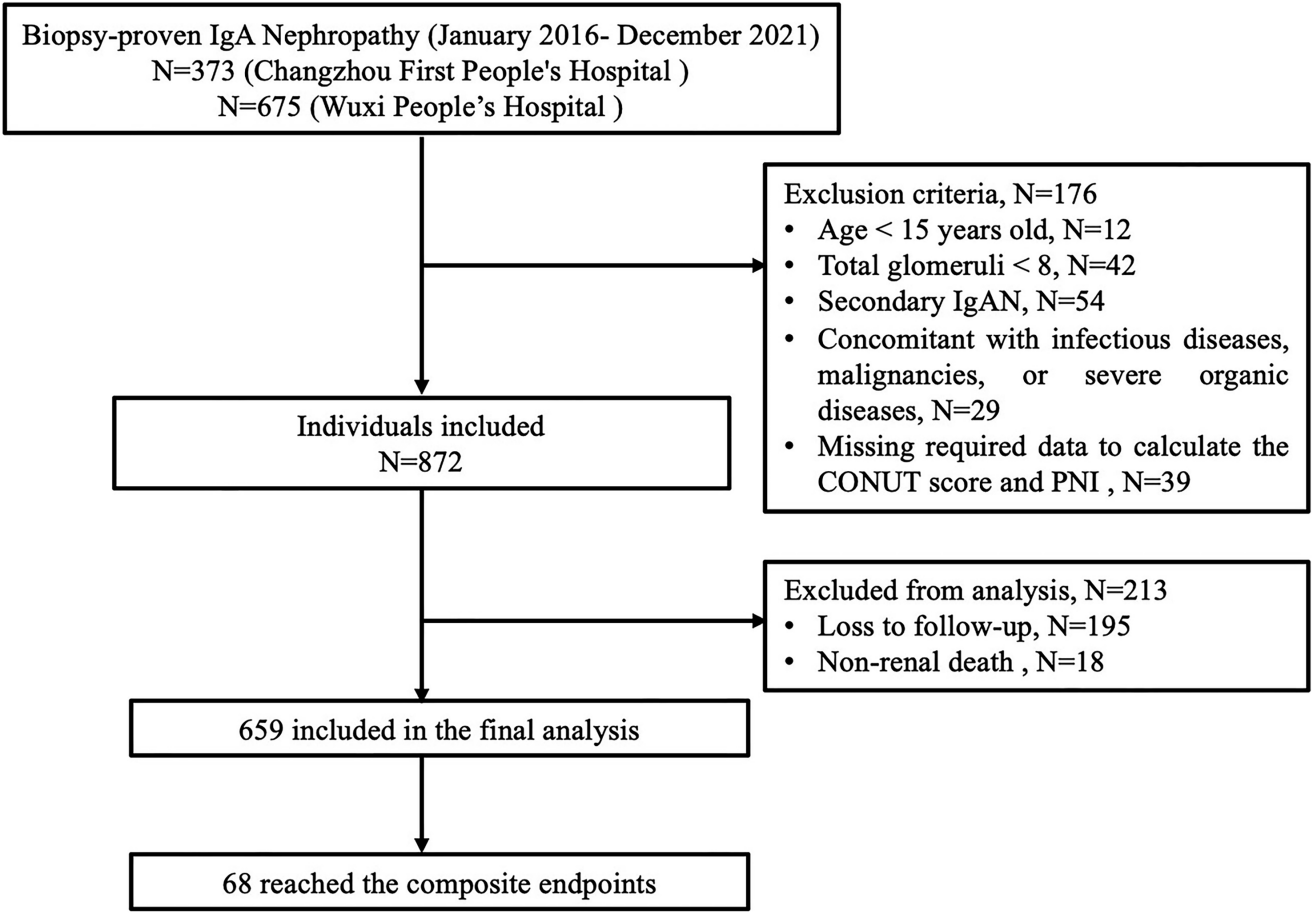
Enrollment and follow-up of study participants.

**Figure 2:**
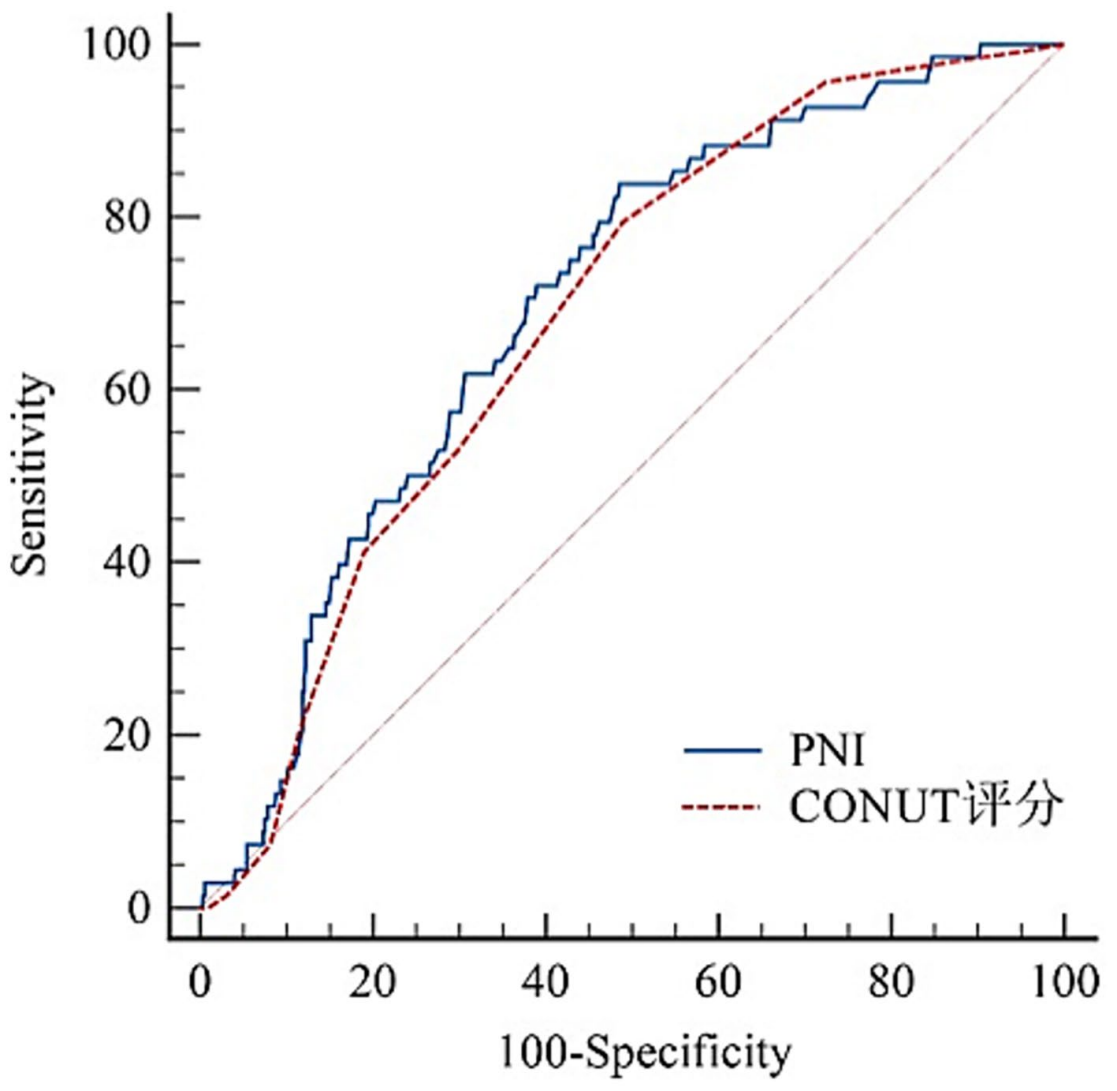
ROC curves of the probability of CONUT score, and PNI in predicting renal outcomes.

A total of 1,048 consecutive patients with biopsy-proven IgAN were recruited from the two centers. Following the screening process, 659 patients constituted the final study population, as detailed in Figure 1. The median age at IgAN diagnosis was 37.00 (30.00, 48.00) years, with 342 (51.90%) being male. Hypertension was present in 296 patients (44.92%). Prior to renal biopsy, 362 patients (54.93%) had received ACEI/ARB, in contrast to only 23 (3.49%) treated with corticosteroids or immunosuppressants. After diagnosis, the proportion of patients treated with ACEI/ARB and immunosuppressive therapy showed a significant increase, reaching 513 (77.85%) and 345 (52.35%), respectively. According to the ROC curve analysis (Figure 2), the optimal cutoff values were determined to be 46.5 for PNI [AUC = 0.699, 95% confidence intervals (CI) = 0.640–0.758] and 2 for CONUT score (AUC = 0.683, 95% CI = 0.625–0.741). A low PNI (≤46.5) and a high CONUT score (>3) were identified in 344 (52.20%) and 343 (52.05%) patients, respectively.

During a median follow-up period of 45 months, 68 patients (10.32%) reached the composite endpoint. The baseline characteristics were shown in Table 1. Compared to the renal survival group, patients in the non-renal survival group had a significantly higher proportion of males and hypertension, elevated levels of BUN, serum creatinine, uric acid, triglycerides, and 24 h urinary protein, as well as higher CONUT scores. On the contrary, they showed lower PNI, a lower rate of ACEI/ARB treatment and decreased levels of hemoglobin, albumin, eGFR, and serum IgM. Regarding pathological characteristics, non-renal survival group exhibited more severe mesangial hypercellularity (M), tubular atrophy/interstitial fibrosis (T), and a higher proportion of global sclerosis. Conversely, the severity of endocapillary hypercellularity (E) was significantly lower. No statistically significant differences were observed for segmental glomerulosclerosis (S), cellular or fibrocellular crescents (C), and the deposition rates of IgG, IgM, C3, and C1q.

**Table 1:** Baseline clinical characteristics of 659 IgAN patients.

Clinical characteristics	Renal survival group (*N* = 591)	Non-renal survival group (*N* = 68)	*p*-value
Age (years)	36.00 (30.00, 47.00)	42.50 (33.50, 54.00)	0.004
Male (*n*, %)	299 (50.59)	43 (63.24)	0.048
Hypertension (*n*, %)	248 (41.96)	48 (70.59)	<0.001
SBP (mmHg)	131.51 ± 16.76	146.63 ± 21.41	<0.001
DBP (mmHg)	83.52 ± 11.89	91.59 ± 12.91	<0.001
MAP (mmHg)	99.51 ± 12.59	109.94 ± 14.29	<0.001
Hemoglobin (g/L)	131.70 ± 19.93	123.82 ± 19.02	0.002
Lymphocyte count (×10^9^/L)	1.95 (1.60, 2.32)	1.83 (1.37, 2.21)	0.057
Albumin(g/L)	36.70 (33.10, 39.60)	32.95 (29.58, 35.92)	<0.001
Globulin (g/L)	26.85 ± 4.15	27.03 ± 4.24	0.737
Fasting blood glucose (mmol/L)	4.66 (4.36, 5.08)	4.66 (4.34, 4.98)	0.489
BUN (mmol/L)	5.54 (4.50, 6.90)	9.55 (7.27, 12.41)	<0.001
Serum creatinine (μmol/L)	90.00 (69.65, 112.00)	165.65 (133.50, 210.05)	<0.001
Uric acid (μmol/L)	370.60 (310.50, 446.70)	449.15 (378.38, 514.42)	<0.001
eGFR (ml/min/1.73m^2^)	83.88 ± 25.73	43.57 ± 19.67	<0.001
Cholesterol (mmol/L)	4.81 (4.17, 5.51)	4.95 (4.24, 5.72)	0.392
Triglyceride (mmol/L)	1.52 (1.07, 2.23)	1.75 (1.30, 2.65)	0.003
Serum IgG (g/L)	10.51 ± 3.04	10.10 ± 3.30	0.298
Serum IgA (g/L)	3.07 (2.40, 3.83)	3.09 (2.07, 4.05)	0.627
Serum IgM (g/L)	1.10 (0.81, 1.52)	0.97 (0.71, 1.35)	0.043
Serum C3 (g/L)	0.89 (0.77, 1.02)	0.86 (0.75, 0.96)	0.082
Serum C4 (g/L)	0.22 (0.18, 0.26)	0.23 (0.19, 0.29)	0.052
24 h urinary protein (g/d)	1.74 (0.97, 2.81)	3.04 (2.00, 4.31)	<0.001
URBC (/μl)	121.55 (41.20, 312.95)	100.10 (25.62, 235.30)	0.112
Treatment			
ACEI/ARB therapy (*n*, %)	474 (80.20)	39 (57.35)	<0.001
Immunosuppressive therapy (*n*, %)	304 (51.44)	41 (60.29)	0.166
PNI	46.80 (42.15, 50.33)	42.23 (38.04, 45.56)	<0.001
CONUT	1.00 (0.00, 3.00)	3.00 (2.00, 4.00)	<0.001
Low PNI (*n*, %)	287 (48.56)	57 (83.82)	<0.001
High CONUT (*n*, %)	289 (48.90)	54 (79.41)	<0.001
Oxford classification			
Mesangial hypercellularity (M1)	380 (64.30)	52 (76.47)	0.045
Endocapillary hypercellularity (E1)	195 (32.99)	11 (16.18)	0.005
Segmental glomerulosclerosis (S1)	437 (73.94)	53 (77.94)	0.475
Tubular atrophy/interstitial fibrosis			<0.001
T1	102 (17.26)	19 (27.94)	
T2	20 (3.38)	19 (27.94)	
Cellular or fibrocellular crescents			0.207
C1	258 (43.65)	25 (36.76)	
C2	10 (1.69)	3 (4.41)	
Global sclerosis (%)	16.67 (7.14, 29.41)	42.27 (25.00, 65.58)	<0.001
Immune complex deposition			
IgG (*n*, %)	164 (27.84)	12 (17.91)	0.082
IgM (*n*, %)	336 (58.33)	38 (56.72)	0.8
C3 (*n*, %)	507 (88.02)	57 (85.07)	0.487
C1q (*n*, %)	24 (4.12)	2 (2.99)	0.904

SBP, systolic blood pressure; DBP, diastolic blood pressure; MAP, mean arterial pressure; BUN, blood urea nitrogen; eGFR, estimated glomerular filtration rate; URBC, urine red blood cell; ACEI, angiotensin-converting enzyme inhibitors; ARB, angiotensin receptor blockers; PNI, prognostic nutritional index; CONUT, controlling nutritional status.

**Figure 3:**
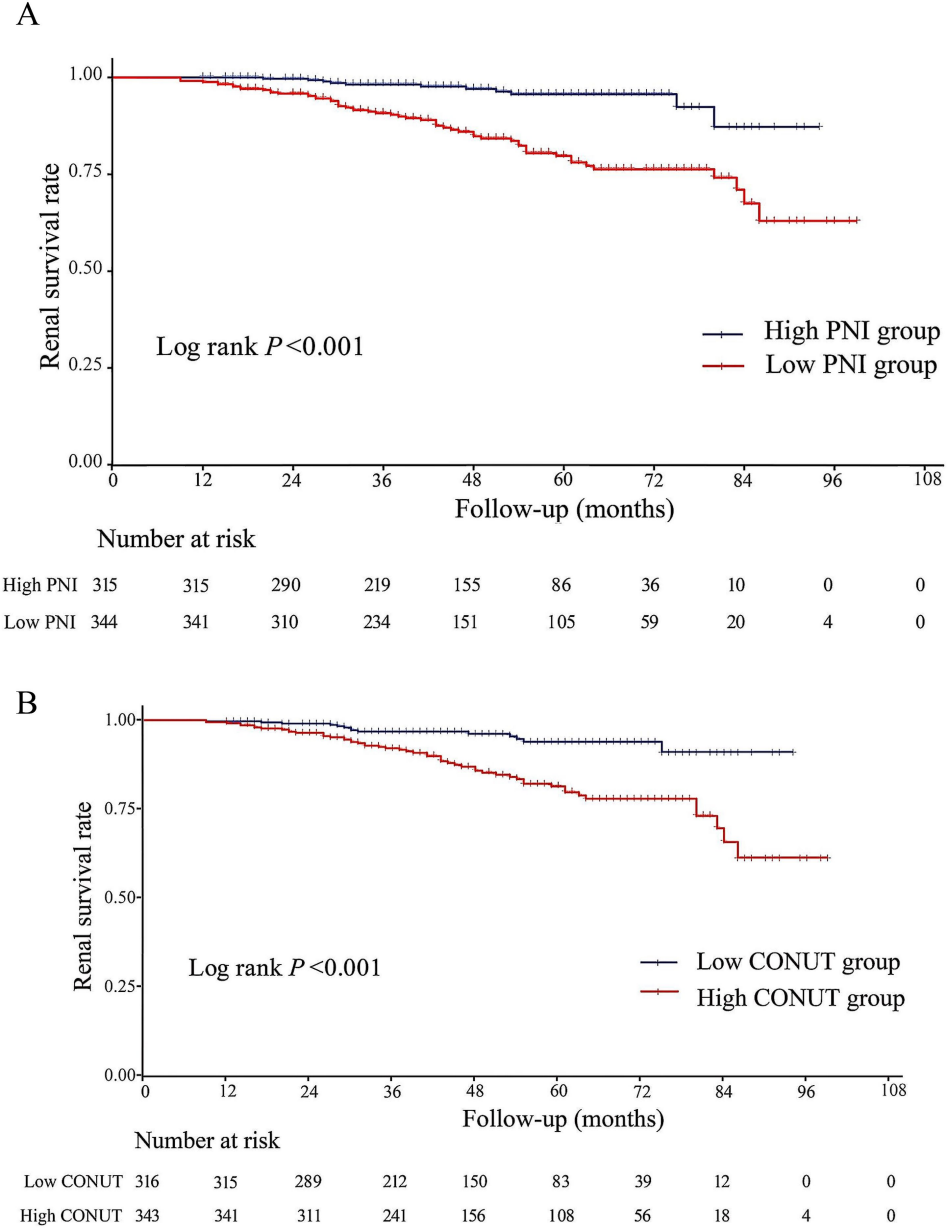
Kaplan–Meier curves for composite renal outcomes in IgAN patients according to objective nutritional indices. **(A)** PNI; **(B)** CONUT score.

The Kaplan–Meier curve revealed that renal-survival rate was significantly higher in high PNI group (Figure 3A, PNI > 46.5; *p* < 0.001) and low CONUT group (Figure 3B; CONUT ≤2; *p* < 0.001). Supplementary Tables S2 and S3 showed the correlations between PNI or CONUT score and selected variables. Univariate Cox regression analysis revealed that both a low PNI (HR = 4.686, 95% CI = 2.455–8.944, *p* < 0.001) and a high CONUT score (HR = 3.445, 95% CI = 1.913–6.203, *p* < 0.001) were significantly associated with increased risk of renal progression. When analyzed as continuous variables, each 1-point increment in the CONUT score increased the risk by 1.209-fold (HR = 1.209, 95% CI = 1.090–1.341, *p* < 0.001); whereas each 1-unit increase in PNI was associated with a 6.7% risk reduction (HR = 0.933, 95% CI = 0.905–0.962, *p* < 0.001). In addition, male, age, hypertension, MAP, hemoglobin, lymphocyte counts, albumin, BUN, serum creatinine, uric acid, eGFR, triglycerides, 24 h urinary protein, E score, T score, and ACEI/ARB therapy were also correlated with renal prognosis (Table 2). Multivariate Cox regression model indicated that low PNI (Table 3; HR = 2.514, 95% CI = 1.212–5.215, *p* = 0.013) and high CONUT (Table 4; HR = 2.152, 95% CI = 1.087–4.259, *p* = 0.028) remained as independent risk factors for poor renal outcomes even after adjustment of these factors. Furthermore, MAP and triglycerides were identified as independent risk factors for renal progression, while eGFR and E-score served as independent protective factors.

**Table 2:** Univariate Cox analysis of composite renal outcomes in IgAN patients.

Variables	Univariate analysis	
	HR (95% CI)	*p*-value
Gender (male vs. female)	1.678 (1.024–2.751)	0.040
Age (year)	1.030 (1.011–1.049)	0.001
Hypertension	3.153 (1.870–5.314)	<0.001
SBP (mmHg)	1.038 (1.027–1.050)	<0.001
DBP (mmHg)	1.043 (1.026–1.060)	<0.001
MAP (mmHg)	1.049 (1.033–1.064)	<0.001
Hemoglobin (g/L)	0.980 (0.968–0.992)	0.001
Lymphocyte count (×10^9^/L)	0.639 (0.412–0.992)	0.046
Albumin(g/L)	0.936 (0.907–0.966)	<0.001
Globumin (g/L)	1.020 (0.966–1.076)	0.478
BUN (mmol/L)	1.422 (1.345–1.504)	<0.001
Serum creatinine (μmol/L)	1.027 (1.023–1.031)	<0.001
Uric acid (μmol/L)	1.006 (1.004–1.008)	<0.001
eGFR(ml/min/1.73m^2^)	0.930 (0.916–0.944)	<0.001
Cholesterol (mmol/L)	1.109 (0.961–1.280)	0.156
Triglyceride (mmol/L)	1.244 (1.072–1.443)	0.004
24 h urinary protein (g/d)	1.108 (1.039–1.182)	0.002
URBC(/μl)	1.000 (0.999–1.000)	0.386
ACEI/ARB therapy	0.421 (0.260–0.682)	<0.001
Immunosuppressive therapy	1.152 (0.707–1.875)	0.570
Oxford classification		
M1	1.393 (0.793–2.447)	0.249
E1	0.414 (0.217–0.791)	0.008
S1	1.323 (0.745–2.347)	0.339
T1 vs. T0	2.465 (1.385–4.388)	0.002
T2 vs. T0	9.330 (5.246–16.591)	<0.001
C1 vs. C0	0.770 (0.467–1.269)	0.304
C2 vs. C0	1.505 (0.463–4.887)	0.497
PNI	0.933 (0.905–0.962)	<0.001
CONUT	1.209 (1.090–1.341)	<0.001
Low PNI	4.686 (2.455–8.944)	<0.001
High CONUT	3.445 (1.913–6.203)	<0.001

SBP, systolic blood pressure; DBP, diastolic blood pressure; MAP, mean arterial pressure; BUN, blood urea nitrogen; eGFR, estimated glomerular filtration rate; URBC, urine red blood cell; ACEI, angiotensin-converting enzyme inhibitors; ARB, angiotensin receptor blockers; PNI, prognostic nutritional index; CONUT, controlling nutritional status.

**Table 3:** Hazard Ratios of PNI for composite renal outcomes in multivariate Cox analysis.

Variables	Multivariate analysis	
	HR (95% CI)	*p-*value
Gender (male vs. female)	1.286 (0.722–2.289)	0.394
Age (year)	0.972 (0.949–0.995)	0.017
Hypertension	1.317 (0.688–2.522)	0.406
Hemoglobin (g/L)	0.991 (0.978–1.004)	0.189
Uric acid (μmol/L)	1.002 (0.999–1.004)	0.233
eGFR(ml/min/1.73m^2^)	0.940 (0.923–0.957)	<0.001
Triglyceride (mmol/L)	1.125 (0.959–1.319)	0.148
24 h urinary protein (g/d)	1.011 (1.924–1.107)	0.810
ACEI/ARB therapy	0.781 (0.439–1.387)	0.398
E1 vs. E0	0.270 (0.131–0.556)	<0.001
T1 vs. T0	0.652 (0.347–1.226)	0.184
T2 vs. T0	1.313 (0.666–2.588)	0.432
Low PNI	2.514 (1.212–5.215)	0.013

eGFR, estimated glomerular filtration rate; ACEI, angiotensin-converting enzyme inhibitors; ARB, angiotensin receptor blockers; PNI, prognostic nutritional index.

**Table 4:** Hazard Ratios of CONUT score for composite renal outcomes in multivariate Cox analysis.

Variables	Multivariate analysis	
	HR (95% CI)	*p-*value
Gender (male vs. female)	1.168 (0.653–2.090)	0.601
Age (year)	0.978 (0.956–1.001)	0.059
Hypertension	1.318 (0.697–2.492)	0.396
Hemoglobin (g/L)	0.992 (0.979–1.006)	0.272
Uric acid (μmol/L)	1.002 (0.999–1.004)	0.213
eGFR(ml/min/1.73m^2^)	0.938 (0.921–0.955)	<0.001
Triglyceride (mmol/L)	1.167 (0.988–1.378)	0.070
24 h urinary protein (g/d)	0.996 (0.912–1.008)	0.933
ACEI/ARB therapy	0.700 (0.397–1.233)	0.216
E1 vs. E0	0.295 (0.145–0.599)	<0.001
T1 vs. T0	0.664 (0.355–1.243)	0.200
T2 vs. T0	1.217 (0.613–2.416)	0.575
High CONUT	2.512 (1.087–4.259)	0.028

eGFR, estimated glomerular filtration rate; ACEI, angiotensin-converting enzyme inhibitors; ARB, angiotensin receptor blockers; CONUT, controlling nutritional status.

**Figure 4:**
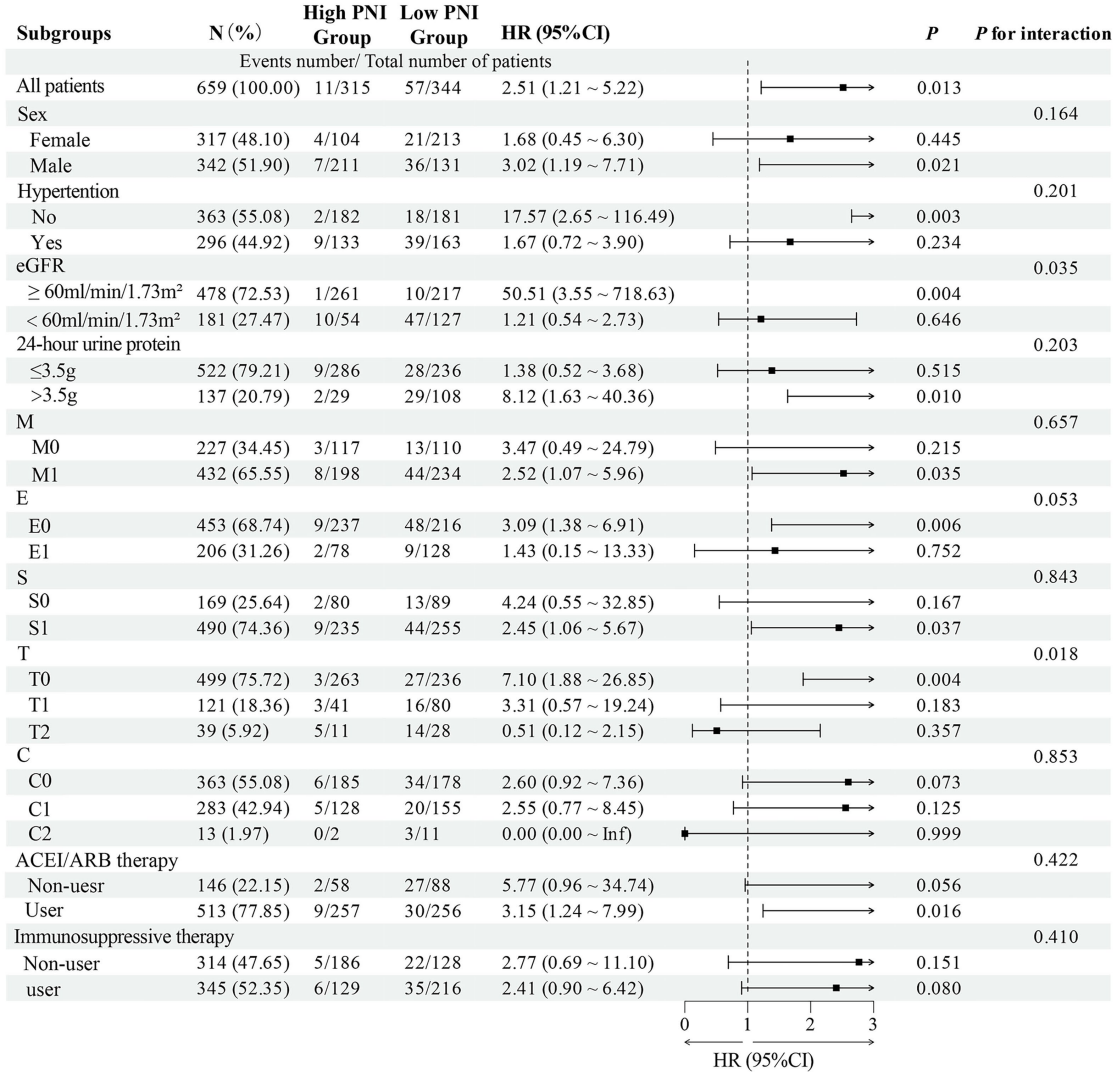
Subgroup analysis and forest plot for the association between the PNI and renal outcomes in IgAN patients.

**Figure 5:**
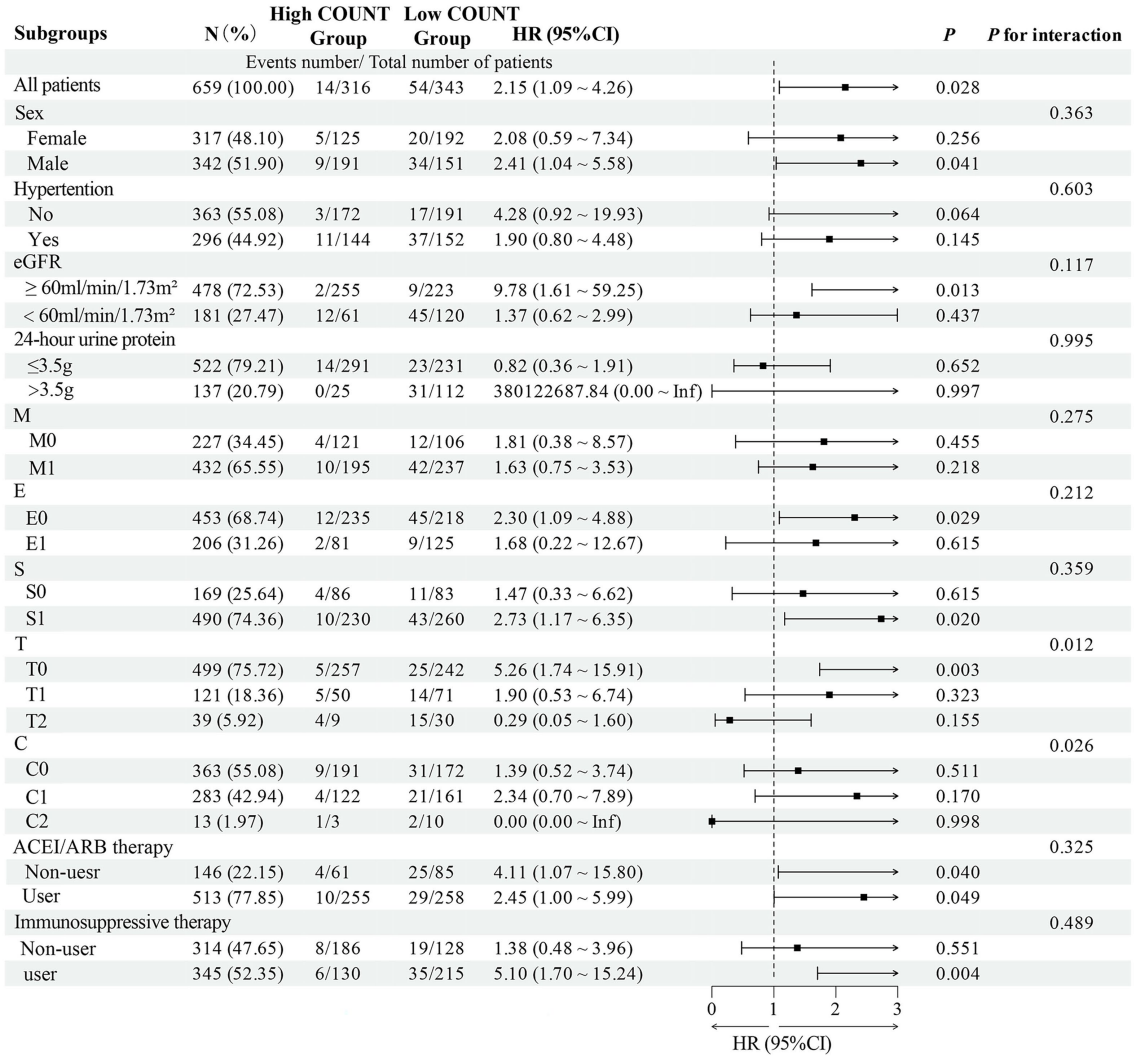
Subgroup analysis and forest plot for the association between the CONUT score and renal outcomes in IgAN patients.

Subgroup analysis revealed significant effect modifications. The association between baseline categorical PNI and renal outcomes exhibited significant heterogeneity across eGFR and T-score subgroups (Figure 4; *p* for interaction < 0.05). Similarly, a significant interaction was observed for the CONUT score within the T-score subgroups (Figure 5; *p* for interaction < 0.05). In the T0 subgroup, both low PNI (HR = 7.10, 95% CI = 1.88–26.85, *p* = 0.004) and high CONUT score (HR = 5.26, 95% CI = 1.74–15.91, *p* = 0.003) were independently associated with poor renal prognosis, unlike in the T1/T2 subgroups. Similarly, low PNI (HR = 50.51, 95% CI = 3.55–718.63, *p* = 0.004) and high CONUT score (HR = 9.78, 95% CI = 1.61–59.25, *p* = 0.013) significantly predicted poor outcomes in eGFR≥60 mL/min/1.73m^2^ subgroup but not in eGFR<60 mL/min/1.73m^2^ subgroup.

## Discussion

In the present study, PNI and CONUT score were prognostic indicators with area under the ROC curve (AUC) of 0.699 and 0.683 in biopsy-proven IgAN patients, respectively. Furthermore, we observed that both a low PNI and a high CONUT score remained independently associated with an increased risk of composite renal outcomes in IgAN after adjustment for relevant clinicopathological confounders.

Objective nutritional indices such as PNI, CONUT score, have been increasingly recognized in various clinical contexts (17, 19). Recent years have witnessed a growing interest in the assessment of nutritional indices to CKD (20, 21). However, evidence regarding the association between these nutritional indices and renal outcomes in IgAN patients is still limited. We observed that patients who reached the composite renal outcomes had lower baseline PNI and higher CONUT score, demonstrating poorer nutritional status among these patients at renal biopsy. Qian et al. found patients with ESKD showed significantly lower body mass index (BMI), PNI, and geriatric nutritional risk index (GNRI), with higher CONUT score. Nevertheless, only GNRI emerged as an independent risk factor for ESKD in multivariable Cox regression model (11). Consistent with our findings, IgAN patients exhibiting poorer nutritional status at renal biopsy are at a substantially increased risk of renal function progression. The GNRI is calculated as follows: 14.89 × serum albumin (g/dL) + 41.7 × body weight/ideal body weight (22). It is noteworthy that body weight is a key component in the GNRI calculation; however, it can be significantly influenced by fluid retention, particularly among patients presenting with nephrotic syndrome. Body weight data were incomplete for some patients. Given these considerations, the prognostic value of the GNRI for IgAN patients was not assessed in this study. Cordos et al. demonstrated that Prognostic Inflammatory and Nutritional Index (PINI) was an effective tool for identifying the silent malnutrition-inflammation syndrome and predicting the risk of acute events in the maintenance hemodialysis patients (23). The PINI was calculated using the formula alpha1-Acid Glycoprotein (AGP) × C-reactive protein (CRP)/albumin × transthyretin. Given that AGP, CRP and transthyretin are not part of routine clinical assessments, we aimed to conduct a prospective study to compare these nutritional indices in IgAN patients in the future.

The PNI and CONUT score serve not only as nutritional assessment tools but also as significant biomarkers of systemic inflammatory status. Notably, both indices incorporate albumin and lymphocytes as shared constituents. Serum albumin, a negative acute-phase protein, exhibits a decline in both its circulating levels and antioxidant capacity during systemic inflammation. It has been confirmed as an independent risk factor for progression to ESKD in IgAN patients (24). Lymphocytes promote the development and progression of renal injury and fibrosis by modulating key processes including apoptosis, proliferation, and fibroblast activation. Furthermore, specific T-cell subsets, such as T helper 2 cells (Th2) and CD8+ T cells, have been noted to play dual roles in CKD (25). Hence, in a non-inflammatory setting, lymphocyte counts reflect the immune status of CKD patients to a certain extent. These mechanisms collectively illuminate the multifaceted nature of the PNI and CONUT score in assessing nutritional and inflammatory status. Furthermore, Gut-kidney axis plays an important role in the IgAN pathogenesis. Malnutrition not only impairs the reparative capacity of intestinal epithelial cells to compromise the gut barrier function, but also induces gut microbiota dysbiosis (26). This dysbiosis, characterized by a diminution of beneficial bacteria and a proliferation of pathogenic species, further exacerbates barrier disruption (27). Compromised intestinal barrier function can lead to dysregulation of the gut mucosal immune system, thereby increasing the production of galactose-deficient IgA1 (Gd-IgA1). These aberrant molecules form circulating immune complexes, which deposit in the glomerular mesangium, triggering inflammation and renal injury (13). Additionally, malnutrition is linked to chronic low-grade inflammation, marked by elevated inflammatory cytokines such as interleukin-6 and C-reactive protein, which in turn promote mesangial cell proliferation, extracellular matrix expansion, and tubulointerstitial fibrosis (28). The depletion of antioxidant defenses and the consequent rise in oxidative stress due to malnutrition exacerbate renal injury as well (29). These potential mechanisms may partially account of the observed association between malnutrition and adverse outcomes in IgAN patients.

To the best of our knowledge, it is the first study to demonstrate that patients with high CONUT score had severe renal outcomes. Total cholesterol is another key component of CONUT score. Dyslipidemia is associated with kidney damage. Li W et al. found that total cholesterol to high-density lipoprotein cholesterol ratio had predictive value in CKD progression (30). Moreover, our finding aligns with and strengthens the existing evidence that the PNI is an independent risk factor for the renal progression to renal failure in IgAN patients (31). The PNI was also identified as a novel acute kidney injury predictor in IgAN across diverse prediction models (32). However, the optimal cutoff values require validation in future studies. To better understand the value of CONUT score and PNI in predicting renal prognosis, we conducted subgroup analysis. In the T0 and eGFR≥60 mL/min/1.73m^2^ subgroup, both low PNI and high CONUT score were independent risk factors for renal progression in IgAN patients, whereas no significant associations were observed in patients with T1/T2 or eGFR<60 mL/min/1.73m^2^. This suggested that improving nutritional status in the early stages may be more effective in delaying renal progression. Given the modest number of events, these findings should be interpreted with caution due to the potential for statistical instability and overfitting. In addition, external validation and prospective interventional studies are warranted to determine whether improving PNI or CONUT scores can lead to better renal outcomes.

Our study also identified eGFR as an independent protective factor for renal progression in IgAN, which is entirely consistent with its role in the International IgAN Prediction Tool (6). Contrary to the International IgAN Prediction Tool, we found E1 was an independent protective factor for renal progression in IgAN. In a sensitivity analysis excluding patients with immunosuppressive therapy, the E-score did not retain a significant association with the risk of renal progression (HR = 0.464, 95% CI = 0.139–1.542, *p* = 0.210). Of note, the E-score has not been reliably linked to the prediction of poor renal outcomes in existing literatures (33). We speculated this finding was driven by a treatment bias. Patients with E1 were more likely to receive immunosuppressive therapy, which are known to reverse active glomerular lesions and thereby improve renal survival (34). Thus, immunosuppressive therapy may have masked the true prognostic value of the E-score. This critical insight underscores that any clinical implications of the E-score must account for the effects of treatment.

Several limitations must be considered when interpreting our results. First, the retrospective design may introduce selection bias and residual confounding, potentially restricting the generalizability of our findings. Future large-scale, prospective studies are needed to address these limitations. Given its observational design, our study does not support causal inferences, and any mechanistic interpretations remain speculative. Accordingly, experimental studies or randomized controlled trials are required to establish causality and elucidate the underlying mechanisms. Second, IgAN is a slowly progressing disease, the median follow-up period of 45 months was relatively short. We are conducting ongoing prospective follow-up of this cohort to validate the long-term prognostic utility of these biomarkers. Furthermore, potential treatment confounding should be considered. Additionally, the nutritional assessment was based solely on a single measurement of CONUT score and PNI at the time of renal biopsy, lacking longitudinal data to track their dynamic changes. Future studies incorporating serial measurements and comparisons with direct inflammatory markers are warranted to elucidate the impact of their temporal changes on renal outcomes and to confirm the robustness of the findings.

## Conclusion

The present study demonstrates that both low PNI and high CONUT score are independently associated with an increased risk of renal progression in IgAN patients with CKD stages 1–4, highlighting their potential utility as prognostic markers. Given their low cost and straightforward calculation, these objective nutritional indices can be seamlessly integrated into clinical practice for clinicians to optimize risk assessment and guide informed decisions on personalized treatment plans.

## Data Availability

The original contributions presented in the study are included in the article/Supplementary material, further inquiries can be directed to the corresponding authors.
