# Supplementary material for: Evaluation of objective nutritional indices as predictors of renal progression in IgA nephropathy patients
Source: Front Nutr. 2026 May 13;13:1773914. doi: 10.3389/fnut.2026.1773914 (PMC13212076; doi:10.3389/fnut.2026.1773914)
Supplement: Supplementary file 1 [file Table_1.docx]

Table S1. Evaluation of CONUT scores

|  | Degree of malnutrition | | | |
| --- | --- | --- | --- | --- |
| Parameter | Normal | Mild | Moderate | Severe |
| Serum albumin (g/L) | ≥35 | 30-34.9 | 25-29.9 | <25 |
| Albumin score | 0 | 2 | 4 | 6 |
| Total lymphocytes (count/mm^3^) | ≥1600 | 1200-1599 | 800-1199 | <800 |
| Lymphocytes score | 0 | 1 | 2 | 3 |
| Total cholesterol (mmol/L) | >4.65 | 3.62-4.65 | 2.58-3.62 | <2.58 |
| Cholesterol score | 0 | 1 | 2 | 3 |
| CONUT scores | 0-1 | 2-4 | 5-8 | 9-12 |

CONUT: Controlling Nutritional Status

Table S2. Correlation analysis between PNI and variables

| Variables | Correlation coefficient (*r*) | *P-*value |
| --- | --- | --- |
| Age (year) | -0.226 | <0.001 |
| SBP (mmHg) | -0.049 | 0.211 |
| DBP (mmHg) | 0.043 | 0.273 |
| MAP (mmHg) | 0.003 | 0.946 |
| Hemoglobin (g/L) | 0.512 | <0.001 |
| Lymphocyte count (×10^9^/L) | 0.497 | <0.001 |
| Albumin (g/L) | 0.881 | <0.001 |
| Globulin (g/L) | 0.211 | <0.001 |
| BUN (mmol/L) | -0.089 | 0.022 |
| Serum creatinine (µmol/L) | -0.082 | 0.034 |
| Uric acid (µmol/L) | 0.088 | 0.024 |
| eGFR(ml/min/1.73m^2^) | 0.236 | <0.001 |
| Cholesterol (mmol/L) | -0.129 | 0.001 |
| Triglyceride (mmol/L) | 0.035 | 0.372 |
| Serum IgG (g/L) | 0.283 | <0.001 |
| Serum IgA (g/L) | 0.144 | <0.001 |
| Serum IgM (g/L) | -0.032 | 0.417 |
| Serum C3 (g/L) | 0.235 | <0.001 |
| Serum C4 (g/L) | 0.019 | 0.625 |
| 24h urinary protein (g/d) | -0.44 | <0.001 |
| Oxford classification |  |  |
| Mesangial hypercellularity | -0.07 | 0.073 |
| Endocapillary hypercellularity | -0.134 | 0.001 |
| Segmental glomerulosclerosis | -0.005 | 0.906 |
| Tubular atrophy/interstitial fibrosis | -0.181 | <0.001 |
| Cellular or fibrocellular crescents | -0.104 | 0.007 |

SBP, systolic blood pressure; DBP, diastolic blood pressure; MAP, mean arterial pressure; BUN, blood urea nitrogen; eGFR, estimated glomerular filtration rate; PNI, prognostic nutritional index.

Table S3. Correlation analysis between CONUT score and variables

| Variables | Correlation coefficient (*r*) | *P*-value |
| --- | --- | --- |
| Age (year) | 0.112 | 0.004 |
| SBP (mmHg) | 0.026 | 0.511 |
| DBP (mmHg) | -0.072 | 0.064 |
| MAP (mmHg) | -0.036 | 0.36 |
| Hemoglobin (g/L) | -0.418 | <0.001 |
| Lymphocyte count (×10^9^/L) | -0.374 | <0.001 |
| Albumin (g/L) | -0.719 | <0.001 |
| GlobuLin (g/L) | -0.256 | <0.001 |
| BUN (mmol/L) | 0.062 | 0.11 |
| Serum creatinine (µmol/L) | 0.098 | 0.012 |
| Uric acid (µmol/L) | -0.056 | 0.152 |
| eGFR(ml/min/1.73m^2^) | -0.18 | <0.001 |
| Cholesterol (mmol/L) | -0.167 | 0.152 |
| Triglyceride (mmol/L) | -0.093 | 0.017 |
| Serum IgG (g/L) | -0.227 | <0.001 |
| Serum IgA (g/L) | -0.141 | <0.001 |
| Serum IgM (g/L) | 0.013 | 0.745 |
| Serum C3 (g/L) | -0.238 | <0.001 |
| Serum C4 (g/L) | -0.048 | 0.219 |
| 24h urinary protein (g/d) | 0.349 | <0.001 |
| Oxford classification |  |  |
| Mesangial hypercellularity | 0.075 | 0.056 |
| Endocapillary hypercellularity | 0.106 | 0.007 |
| Segmental glomerulosclerosis | -0.028 | 0.481 |
| Tubular atrophy/interstitial fibrosis | 0.171 | <0.001 |
| Cellular or fibrocellular crescents | 0.104 | 0.007 |

SBP, systolic blood pressure; DBP, diastolic blood pressure; MAP, mean arterial pressure; BUN, blood urea nitrogen; eGFR, estimated glomerular filtration rate; CONUT, controlling nutritional status.
